# Novel Biodegradable Polyesters. Synthesis and Application as Drug Carriers for the Preparation of Raloxifene HCl Loaded Nanoparticles

**DOI:** 10.3390/molecules14072410

**Published:** 2009-07-07

**Authors:** Dimitrios Bikiaris, Vassilios Karavelidis, Evangelos Karavas

**Affiliations:** 1 Laboratory of Organic Chemical Technology, Chemistry Department, Aristotle University of Thessaloniki, 541 24 Thessaloniki, Greece; 2 Pharmathen S.A., Pharmaceutical Industry, Dervenakion Str 6, Pallini Attikis, 153 51 Attiki, Greece

**Keywords:** raloxifene HCl, aliphatic polyesters, block copolymers, biodegradable polymers, nanoparticles

## Abstract

Raloxifene HCl is a drug with poor bioavailability and poor water solubility. Furthermore nο pharmaceutically acceptable organic solvent has been reported before to dilute the drug. It was observed that Raloxifene HCl can be diluted in a solvent mixture of acetone/water or ethanol/water. The aim of this study was to use biodegradable polymers in order to prepare Raloxifene HCl nanoparticles. For this purpose a series of novel biodegradable poly(ethylene succinate-co-propylene adipate) P(ESu-co-PAd) polyesters were synthesized following the polycondensation method and further, poly(ethylene succinate) (PESu) and poly(propylene adipate) (PPAd) were used. The prepared polyesters were characterized by intrinsic viscosity measurements, end group analysis, enzymatic hydrolysis, Nuclear Magnetic Resonance Spectroscopy (^1^^Η^-NMR and ^13^C-NMR) and Wide-angle X-ray Diffractometry (WAXD). The drug nanoparticles have been prepared by a variation of the co-precipitation method and were studied by Wide-angle X-ray Diffractometry (WAXD), FTIR spectrometry, light scattering size distribution, Scanning Electron Microscopy (SEM) and release behavior measurements. The interactions between the polymers and the drug seem to be limited, so the drug occurs in crystalline form in all nanoparticles. The size of the nanoparticles seems to be in the range of 150-350 nm, depending on the polymer that was used. The drug release depends on the melting point and degree of crystallinity of the polyesters used. An initial high release rate was recorded followed by very slow rates of controlled release.

## 1. Introduction

Aliphatic polyesters, due to their favorable features of biodegradability and biocompatibility, constitute one of the most important classes of synthetic biodegradable polymers and are nowadays available commercially in a variety of types. Some examples of aliphatic polyesters one may mention are polycaprolactone (PCL), poly(hydroxybutyrate) (PHB), poly(3-hydroxybutyrate-co-3-hydroxy-valerate) (Biopol^®^), poly(L-lactide), etc., [1,2,3,4]. Most of these polyesters have been studied for their biocompatibility, bioresorbability and their cytocompatibility as well [5, 6]. It was found that they are biocompatible materials with higher hydrolysability into human body and therefore they can be used as drug carriers for controlled release devices and for biomedical applications. Targeting drug delivery systems have been studied widely in cancer therapeutic applications [7,8,9,10,11]. In the last years poly(alkylene dicarboxylates) such as poly(propylene succinate) (PPSu) and poly(propylene adipate) (PPAd) have been synthesized and studied [12,13,14,15,16,17,18,19]. These polyesters are appropriate for medical and biomedical applications including drug delivery systems by preparing drug loaded nanoparticles or solid dispersions. Only a few studies have been reported so far for the preparation of nanoparticles and solid dispersions for drug delivery systems using such aliphatic polyesters [20].

**Figure 1:**
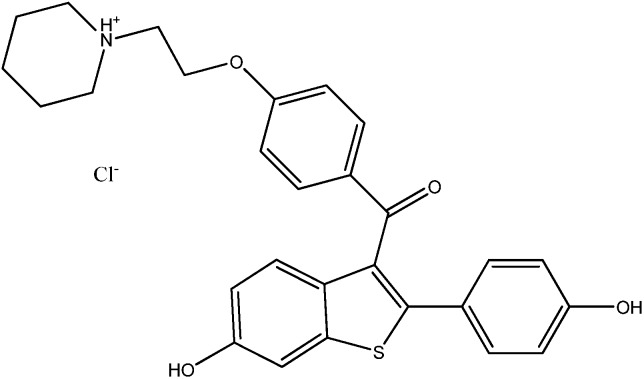
Chemical structure of Raloxifene HCl.

Raloxifene HCl (Figure 1) is a polyhydroxylated non-steroidal compound with a benzothiophene core. It is an estrogen agonist in bone, where it exerts an antiresorptive effect. The results of several large clinical trials have shown that raloxifene reduces the rate of bone loss at both distal sites and in the spinal column and may increase bone mass at certain sites [21]. The drug has beneficial actions on lipoprotein metabolism, reducing both total cholesterol and LDL; however, HDL is not increased unlike with estrogen-replacement therapy. Pre-clinical studies indicate that raloxifene has an antiproliferactive effect on estrogen receptor (ER)-positive breast tumors and on the proliferation of ER-positive breast cancer cell lines and significantly reduces the risk of ER-positive but not ER-negative breast cancer [22]. Adverse effects include deep vein thrombosis, pulmonary embolism and leg cramps [23]. 

Raloxifene HCl is a generic name for [6-hydroxy-2-(4-hydroxyphenyl)benzo-[b]thien-3-yl][4-[2-(1-piperidinyl)ethoxy]-phenyl]ethanone hydrochloride with a molecular weight of 510.05 g∙mol^−1^. It is off-white to pale yellow non-volatile solid. Its solubility in water is 627.4±132.0 μg∙mL^−1^ and it is classified as very slightly soluble in water [24]. Raloxifene is adsorbed rapidly after oral administration and has an absolute bioavailability of about 2 percent. The drug has a half-life of about 28 h and is eliminated primarily in the faeces after hepatic glucuronidation [23]. The drug exhibits high interindividual and intraindividual variability (30 percent) of most pharmacokinetic parameters [21] and this fact makes it attractive for further disposition and metabolism studies.

In the present study copolymers related to the aliphatic polyesters poly(ethylene succinate) (PESu) and poly(propylene adipate) (PPAd) were synthesized and applied in preparing drug loaded carriers of raloxifene HCl. The purpose of this study was to prepare raloxifene HCl nanoparticles with coprecipitation method [25] and to evaluate the dissolution behavior of the drug from these nanoparticles. Raloxifene HCl nanoparticles have never been reported before, but their preparation very important because it could reduce the possibility of toxic results for the patients. Furthermore, raloxifene HCl nanoparticles would probably increase the drug’s bioavailability. 

## 2. Results and discussion

### 2.1. Polymer characterization

**Figure 2:**
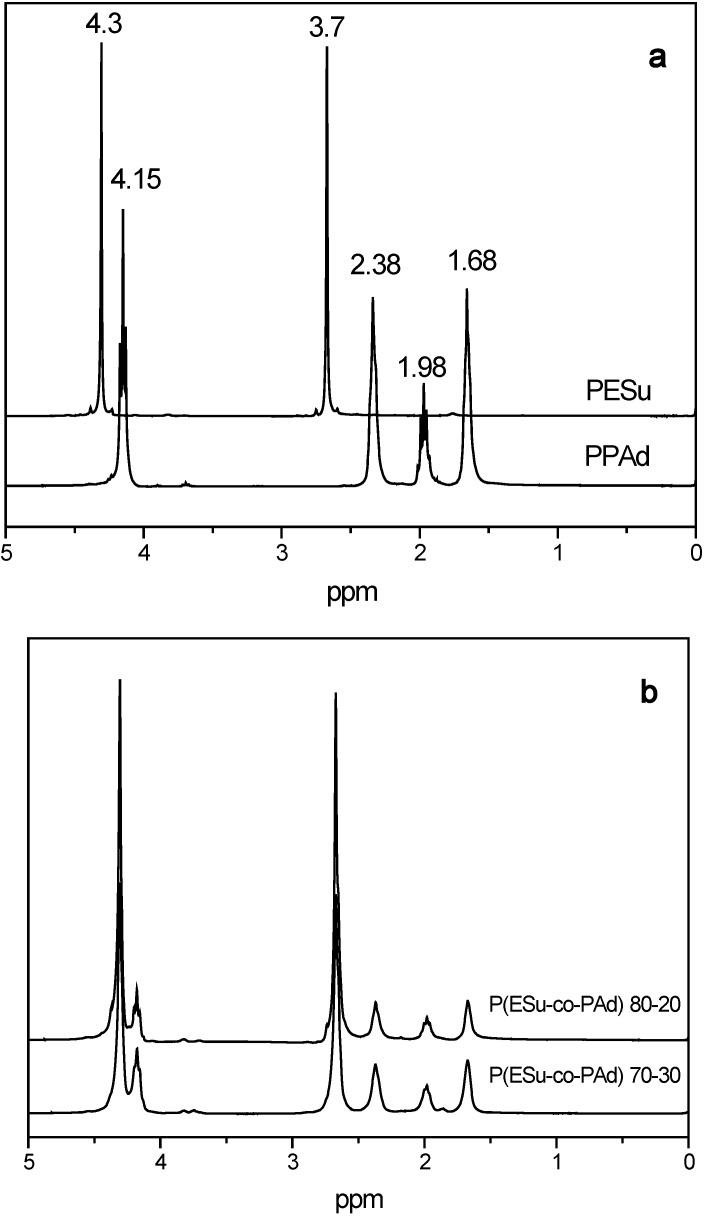
^1^H-NMR spectra of a) PESu, PPAd and b) their P(ESu-co-PAd) co-polymers.

The prepared P(ESu-co-PAd) copolyesters that were used as drug carriers in the present work were studied by ^1^H-NMR, ^13^C-NMR, intrinsic viscosity measurements, WAXD and enzymatic hydrolysis. The I.V. values were in the range of 0.4-0.6 dL/g (Table 1) for all the copolymers. These values were expected due to the extended degradation reaction that takes place at elevated temperatures and are in agreement with the I.V. values of other aliphatic poly(alkylene dicarboxylates) that were synthesized in previous studies [26,27]. Both ^1^H-NMR and ^13^C-NMR spectra have been taken for the homopolymers PESu and PPAd and their co-polymers in order to identify their structures (Figure 2). 

The ^1^H-NMR spectrum of PESu presents characteristic peaks at 7.3, 4.3, 3.7 and 2.68 ppm, while the PPAd spectra present characteristic peaks at 7.3, 4.15, 3.7, 2.38, 1.96 and 1.66 ppm. All these characteristic peaks can be also recorded in the copolymers as well. Furthermore, it can be mentioned that studying the ^1^H-NMR spectra for the copolymers, the intensity of the peaks relative to PPAd increases with increasing the PAd amount in the copolymers. From these peaks the copolymer composition was calculated from the relative areas of ^1^H-NMR resonance peaks of corresponding methylene protons of each polymer. From Table 1 it is obvious that for the prepared copolyesters the actual molar composition is very close to the feed one. A slight increase was observed for propylene adipate sequences. This is maybe due to the lower boiling point of ethylene glycol compared with propylene glycol and thus can be removed easier during polycondansation or due to the higher thermal stability of adipic acid compared with succinic acid [28]. 

**Figure 3:**
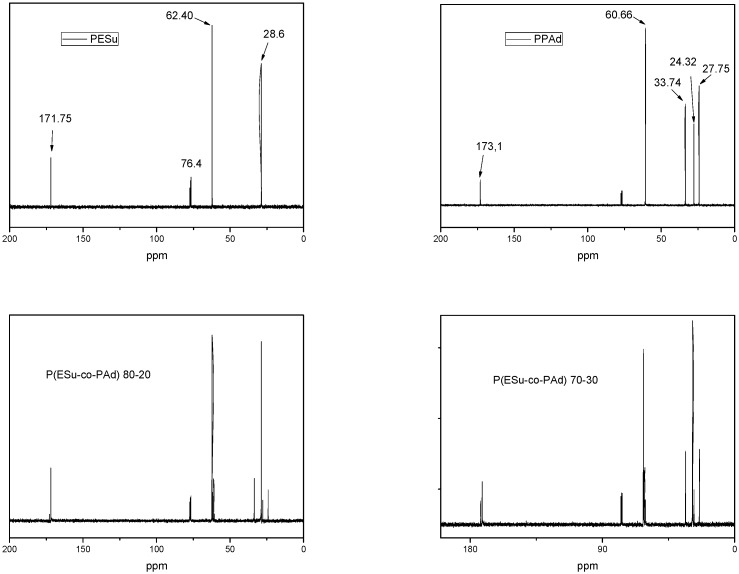
^13^C-NMR spectra of PESu, PPAd and their P(ESu-co-PAd) copolymers.

In order to have a better and clearer view of the synthesized copolyesters the ^13^C-NMR spectra were used in order to quantify and characterize the changes in chain structure as a result of transesterification. The ^13^C-NMR spectra of PESu present characteristic peaks at 171.75, 62.40 and 28.6 ppm and PPAd spectra at 173.1, 60.66, 33.74, 27.75 and 24.32 ppm (Figure 3). As can be seen, all the characteristric peaks of PESu and PPAd are also recorded in their copolymers, as well. The peak at 76.4 ppm may belong to some oligomers. Taking into account these peaks and especially the intensities of –OCH_2 _region that are recorded at 62.40 and 60.66 for PESu and PPAd respectively, it is clear that the prepared P(ESu-co-PAd) copolyesters are random, as was expected according to the procedure used. This was reflected also to their physical state, since the most of the prepared copolymers are amorphous and only three of them are crystalline.

Their melting points have been determined with the usage of a Polar Light Microscope (PLM) and are presented in Table 1 in comparison with the melting points of neat polyesters PESu and PPAd. As can be seen the melting point of P(ESu-co-PAd) copolyesteres decreases by increasing the PAd content, since crystals with less perfection were formed.

**Table 1:** Characteristics of prepared P(ESu-co-PAd) copolymers.

Polymer	PESu/PPAd Feed	^1^H-NMR	[η] (dL/g)	Tm (^o^C)	-COOH (eq/10^6^g)
PESu	100/0	100/0	0.28	120	65
P(ESu-co-PAd) 90/10	90/10	88.9/11.1	0.46	82	54
P(ESu-co-PAd) 80/20	80/20	77.4/22.6	0.50	60	61
P(ESu-co-PAd) 70/30	70/30	67.2/32.8	0.43	35	59
P(ESu-co-PAd) 60/40	60/40	55.6/44.5	0.39	-*	51
P(ESu-co-PAd) 50/50	50/50	46.7/53.3	0.48	-*	58
P(ESu-co-PAd) 40/60	40/60	34.4/65.6	0.50	-*	67
P(ESu-co-PAd) 30/70	30/70	27.3/72.7	0.52	-*	49
P(ESu-co-PAd) 20/80	20/80	18.7/81.3	0.53	-*	43
P(ESu-co-PAd) 10/90	10/90	9.1/90.9	0.62	-*	37
PPAd	0/100	0/100	0.58	43	40

*amorphous material.

For PAd contents higher than 40 percentage of weight all the prepared copolymers are completely amorphous. Additionally, it must be mentioned that PPAd and P(ESu-co-PAd) 70/30 can be characterized as thermosensitive polymers, appropriate as drug carriers in drug delivery systems, because their melting points are near to the range of human’s body temperature. Furthermore, such thermosensitive polymers may result in useful materials in targeting drug delivery systems in cancer therapy [29].

**Figure 4:**
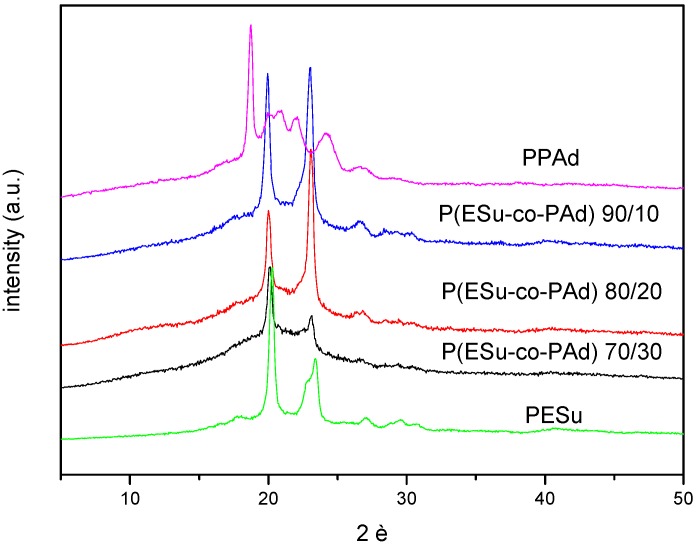
WAXD patterns of the PESu, PPAd and crystalline P(ESu-co-PAd) copolymers.

WAXD was used to examine the crystal structure of the polymers. In Figure 4, indicative WAXD patterns of the neat polyesters and their P(ESu-co-PAd) copolymers are presented. PPAd has characteristic peaks at 2θ= 18.75, 20.04, 20.84, 22.01, 24.19 and 26.71deg. The PESu pattern observed in this work has characteristic peaks at 2θ= 17.88, 20.28, 23.40, 27.08 and 29.6deg corresponding to the known α-crystal forms of these polymers. The P(ESu-co-PAd) 90/10, 80/20 and 70/30 copolymers were also in crystalline form, since all the others with higher content in PAd were amorphous. All polyesters showed characteristic peaks at 2θ=20, 23 and 26deg which is an indication that only PESu moieties are crystallized. This is because at these samples ESu units are in excess and crystallizable sequences can occur along the chain of these copolymers. Furthermore, examining these patterns it can be concluded that the P(ESu-co-PAd) copolymers have differences in the crystallinity. P(ESu-co-PAd) 90/10 has the highest crystallinity while P(ESu-co-PAd) 70/30 has the lowest. In general the copolymer samples showed an amorphous background, due to the lower crystallinity compared to those of the PESu.

**Figure 5:**
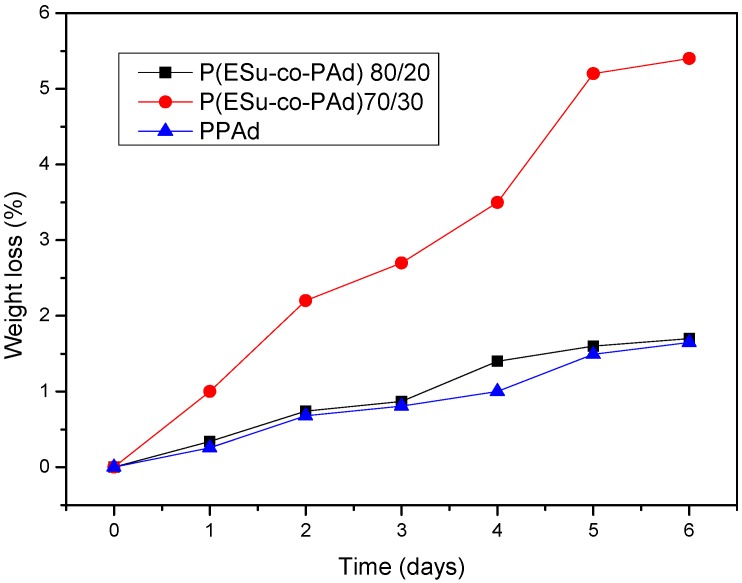
The percentage of mass loss vs time after enzymatic hydrolysis for P(ESu-co-PAd) copolymers.

The enzymatic hydrolysis of the copolymers was studied only for the P(ESu-co-PAd) 90/10 and 80/20 because only these were crystalline enough to prepare films with the desired size and thickness. The profiles of the percentage of mass loss after the enzymatic hydrolysis procedure for the two copolymers are shown in Figure 5. The mentioned copolymers have a weight loss of 2 percent for P(ESu-co-PAd) 90/10 and 5.5 percent for P(ESu-co-PAd) 80/20 in 6 days. This weight loss is much higher compared with neat PPAd. In earlier studies we have realized that biodegradation rates are related to the melting point, the degree of crystallinity and the molecular weight of the polymers. The polyesters with lower melting temperatures and low degree of crystallinity are those that hydrolyze faster. PESu showed low biodegrability during enzymatic hydrolysis due to its high crystallinity. as well as increased melting point. [14]. PPAd in earlier studies showed better biodegradation rates than PESu and PPSu after enzymatic hydrolysis [28]. Figure 5 show that P(ESu-co-PAd) 80/20, which has a lower melting point and crystallinity than P(ESu-co-PAd) 90/10, also degrades faster during enzymatic hydrolysis.

### 2.2. In vitro biocompatibility of aliphatic polyesters

**Figure 6:**
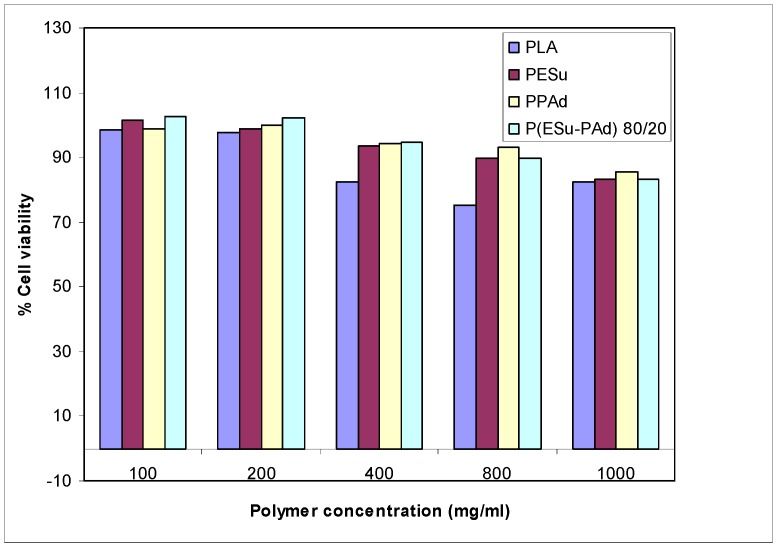
HUVEC cells viability after incubation for 24 hours with different polymers and different polymer concentrations.

The prepared aliphatic P(ESu-co-PAd) copolyesters exhibited low toxicity against HUVEC cells, with appreciable cytotoxicity (higher than 20 percent reduction of cell viability) being observed only after exposing the cells to high nanoparticle concentrations, i.e. higher than 800 μg/mL (Figure 6). Based on polymer toxicity on HUVEC cells, the biocompatibility of PESu, PPAd and their copolymers was deemed comparable to the biocompatibility of PLA, which is a polymer of high biocompatibility and is widely used in biomedical applications [30]. For this reason these aliphatic polyesters may be able to be used as drug carriers as the already used extensively PCL or PLA. From our previous study it was found that a similar aliphatic polyester, namely poly(propylene succinate), is appropriate for use as a drug carrier for drug nanoencapsulation or to prepare formulations by melt mixing [20]. 

### 2.3. Solubility of Raloxifene HCl

The most difficult part in preparing nanoparticles of the drug raloxifene HCl were the dissolution efforts using a solvent that is pharmaceutically accepted. The most common organic solvents used in preparing nanoparticles are acetone, ethanol and chloroform. Raloxifene HCl is not soluble in pure acetone, nor ethanol or chloroform. We were able to dilute the drug in a solvent system of acetone/water and ethanol/water as well. That probably happens because raloxifene HCl acts like a salt due to the hydrochloric anion that is included in the drug’s molecule formation. The solubility of raloxifene depends on how much water will be used. The best solvent/water content that dilutes the maximum amount of the drug and the solubility values are shown below:

- In mixture of acetone/water 10/3 v/v the maximum solubility is 30±5 mg/mL with sonicator operation for 1 minute.
- In mixture of ethanol/water 8/2 v/v the maximum solubility is 45±5 mg/mL.

In this study we chose to work with the acetone/water solvent system which was able to dilute the prepared polymers and the drug, as well. We used the smallest amount of water so as to minimize the polymer loss due to recrystallization during the nanoencapsulation process. The ethanol/water solvent system could be probably used in other drug delivery systems such as solid dispersions of the drug in polymer matrices by the solvent evaporation method.

### 2.4. Nanoencapsulation of Raloxifene HCl in aliphatic polyesters

Polyesters like those prepared in the present study have never been reported before in drug nanoencapsulation. Furthermore efforts to prepare raloxifene HCl nanoparticles that probably would increase the drug’s bioavailability, that is only about 2 percent, have ever been reported, so preparing these nanoparticles is very important and could reduce the possibility of toxic results in humans. Earlier studies reported that nanoparticle formulations for oral, intravenous or pulmonary administration can improve the bioavailability of poorly water soluble drugs and therefore lower levels of dosages can be administrated to the patients, reducing the toxicity effects [56]. In this study raloxifene HCl loaded nanoparticles were prepared for intravenous delivery purpose to achieve high bioavailability that probably would reduce the needed dosage level of the drug and therefore the toxicity effects as well.

#### 2.4.1. Characterization of the nanoparticles

Biodegradable polyesters that can be diluted in acetone solvent can be used successfully in nanoencapsulation drug delivery systems. In this work nanoparticles of the drug raloxifene HCl with the polyesters PESu, PPAd and P(ESu-co-PAd), 80/20 and 70/30 copolymers have been prepared. High molecular weight PESu is insoluble in pure acetone, and for this reason in the present study a polyester with low molecular weight was chosen (I.V.=0,28 dL/g). The prepared nanoparticles with raloxifene HCl were characterized with several methods. 

**Figure 7:**
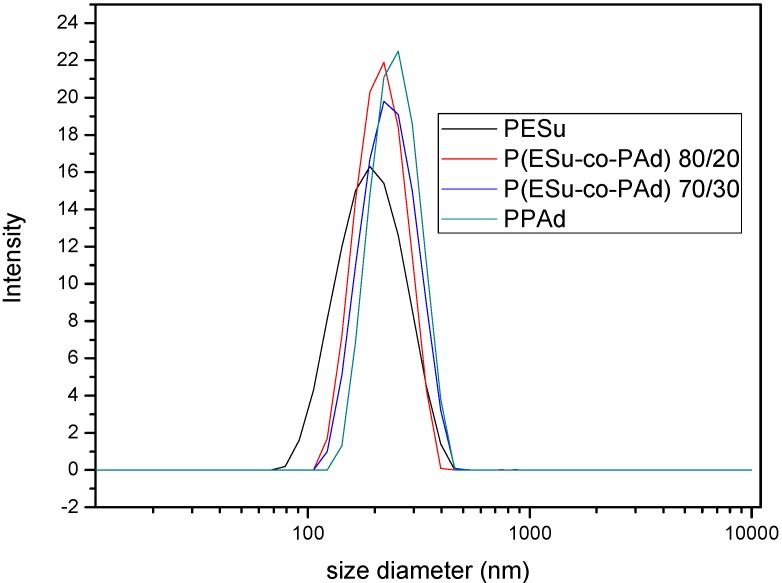
Particle size distribution of Raloxifene HCl loaded nanoparticles.

Particle size distribution was obtained through light scattering and, as it can be seen in Figure 7, the mean diameter is about 200-350 nm. Nanoparticles should have small size to be accepted by human cells. Gaumet *et al*. [31] have reported the claimed sizes of fenestrations of the vasculature in different organs and some pathologies [32,33,34,35,36,37,38,39]. The prepared nanoparticles would be appropriate for tumor drug delivery systems. For bone applications it would be better to prepare nanoparticles with lower particle size (85-150 nm).

**Figure 8:**
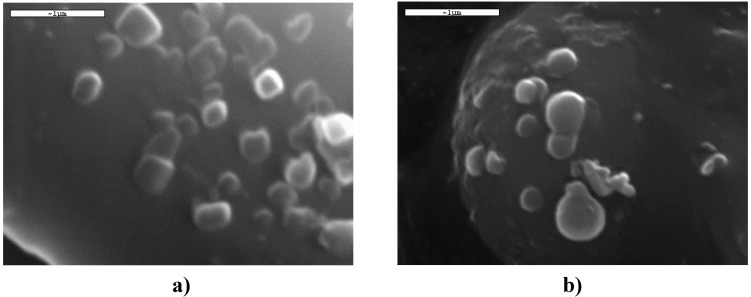
SEM micrographs of the Raloxifene loaded a) P(ESu-co-PAd) 70/30 copolymer and b) PPAd, nanoparticles.

The SEM micrographs of the nanoparticles are shown in Figure 8. It can be seen that the drug-loaded nanoparticles had a discrete spherical shape with a diameter about 250 nm, a fact that is in agreement with the dynamic light scattering measurements. 

Table 2 shows the drug loading characteristics. The percentage of nanoparticle yield seems to be in a good range, which means that the method is well working, with low loss of materials used during the nanoparticle preparation. It was found that the drug loading content was reduced by increasing the Pad amount in the copolymers P(ESu-co-Pad). The percentage of entrapment efficiency showed that raloxifene HCl is inclined to be entrapped in the nanoparticles. That happens because the drug is very hydrophobic and avoids being in touch with the dispersant medium (water) during the nanoparticle preparation. Such high percentage of entrapment efficiency has been reported in earlier studies with poorly water soluble drugs [40]. 

**Table 2:** Average values of nanoparticle yield, drug loading content, entrapment efficiency and particle size of Raloxifene HCl loaded nanoparticles.

Sample	DL (%)	EE (%)	Yield (%)	Mean Diameter (nm)	PdI
PESu	11.73	95	75.6	209	0.29
P(ESu-PAd) 80/20	11.43	92	75.6	279	0.33
P(ESu-PAd) 70/30	9.08	95	72.4	297	0.27
PPAd	7.84	97	64.9	351	0.27

**Figure 9:**
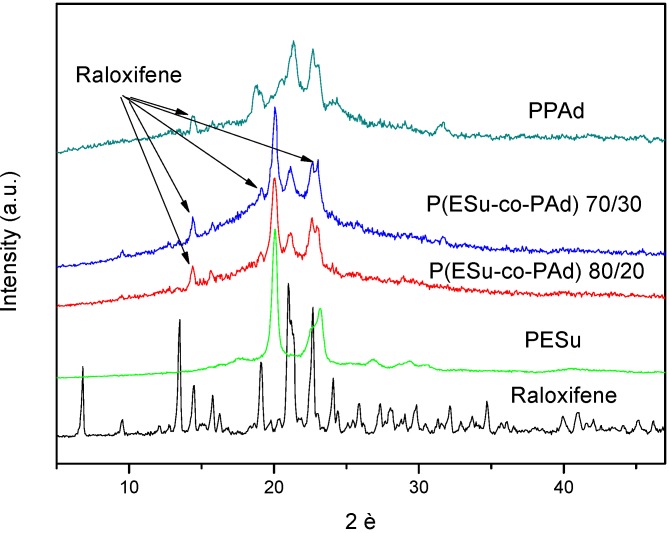
Comparative WAXD patterns of Raloxifene HCl and the drug loaded nanoparticles.

Figure 9 shows the WAXD patterns of the nanoparticles where the characteristic peaks of PESu and these of raloxifene in the nanoparticles can be seen. However, these WAXD patterns revealed that the most intense peaks corresponding to the reflections of the polymer crystalline planes. Raloxifene HCl has characteristic peaks at 2θ= 6.72, 13.70, 14.50, 19.17, 21.01, 22.76 and 24.10 deg. Some peaks are also presented in prepared nanoparticles but at different positions than initial drug. The main peaks are recorded at 2θ= 14.39, 15.81, 19.17 and 23.11 deg which are presented in low magnitude in all samples. This is an indication that the drug is entrapped mainly in a crystalline phase in the polyesters rather that in the amorphous state, but in a different crystalline structure than the initial drug. This is very usual in drugs that are recrystallized from solvents [41,42,43]. Since the size of nanoparticles is lower than 300 nm, it is concluded that the drug is entrapped in polymer nanoparticles in the form of nanocrystals. 

**Figure 10:**
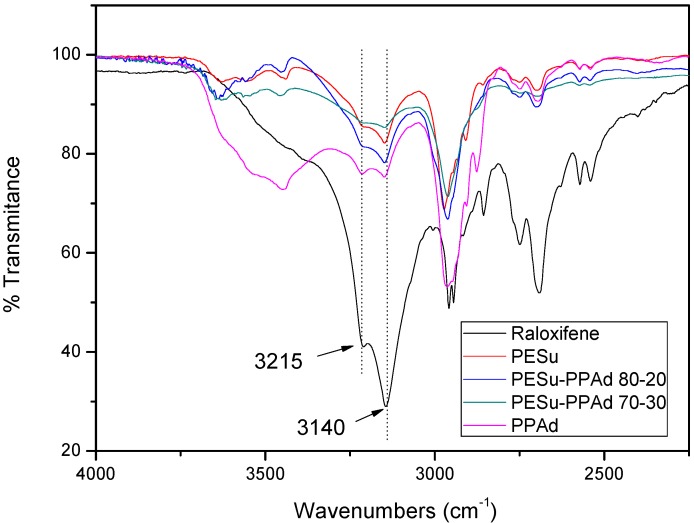
Comparative FTIR spectra of Raloxifene HCl and the drug loaded nanoparticles.

FTIR spectroscopy was used to study the possibility of interactions between the polyesters and raloxifene HCl. Raloxifene HCl has a lot of characteristic peaks at 2,956, 2,946, 2,750, 2,690, 2,570 and 2,540 cm^-1^ but the most important is the double peak at 3,215 and 3,140 cm^-1^ due to its functional N-H and Ph-OH bonds (Figure 10). During the preparation of the nanoparticles any kind of physicochemical interactions that may take place, like the formation of hydrogen bonds between the carriers and drug, will automatically lead to frequency shifts or splitting in absorption peaks. The aliphatic polyester carriers used have a lot of ester or hydroxyl groups that may be able to interact with the above mentioned group of raloxifene. However, as can be seen in all recorded spectra the position of the raloxifene characteristic peaks remain unaffected, which is evidence that such hydrogen bond interactions cannot take place. This could explain why the drug is in crystalline form in all nanoparticles, because it has been reported that interactions between the polymers and the drugs in drug carrier systems lead to amorphization of the drug [44].

#### 2.4.2. Dissolution studies of the nanoparticles

**Figure 11:**
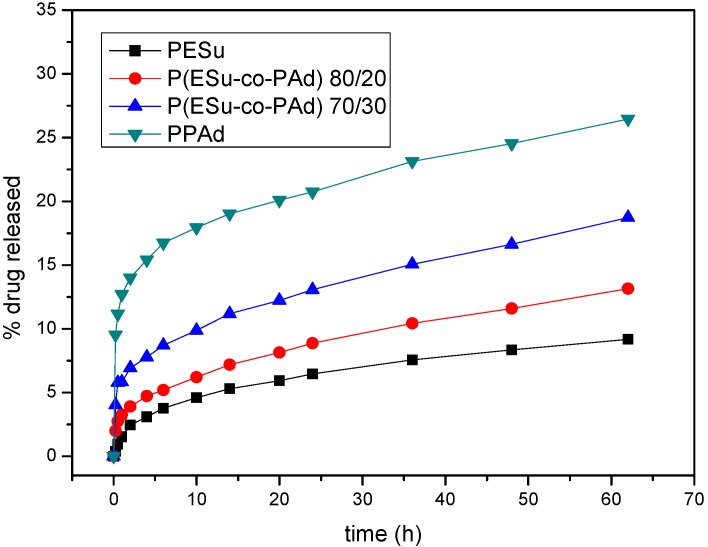
Raloxifene HCl release profiles from the prepared nanoparticles of PESu, PPAd and P(ESu-co-PAd) 80/20 and 70/30 copolymers.

The aim of this study was to prepare raloxifene nanoparticles for intravenous delivery to achieve controlled release with higher bioavailability and reduced toxicity effects in human body. Comparing the nanoparticles that were prepared, only PPAd and P(ESu-co-PAd), which have melting points very near to human body temperature, showed briefly enhanced release rates. All samples showed initially an increased release of the drug that was mainly adsorbed at the polymer matrices surface, followed by a controlled release with low release rates. However, as can be seen from Figure 11, PESu and the P(ESu-co-PAd) 80/20 copolymer, which have the highest melting points, have the lowest release rate at the initial stages. It must be mentioned that the drug loading is not similar for the prepared polyesters. So the release profiles show the percentage of mass of the loaded drug that was released over time, however higher amount of nanoparticles with lower drug loading content is needed to achieve the same mass conditions of the fed drug for the dissolution test of each sample.

The most important factor that affects the dissolution studies is the crystallinity of the nanoparticulate system that is prepared and the insolubility of the prepared polyesters in the dissolution medium. As has been previously reported, the higher the crystallinity of the polymer matrix, the faster the release of the drug [45, 46] a fact that is being explained by the hypothesis that the high crystallinity leads to the formation of microchannel structure [47] and forces the drug to a faster release. However, in our polyesters it seems that the melting point of the drug carrier plays also an important role. As can be seen the polymers with the lowest melting points PPAd and P(ESu-co-PAd) 70/30 have the highest drug release rates. It seems that these polymers behaved as thermosensitive polymers. 

Raloxifene releases slowly from the nanoparticles due to its hydrophobicity and its high crystallinity within the nanoparticles. It seems that the drug forms nanocrystalls within the nanoparticles and so it is difficult for the dissolution medium to move the drug throughout the microchannel structure which occurs due to the polyester matrices that are in high crystalline form. The diffusion of the drug nanocrystalls seems to be very difficult. Furthermore it has been realized that the higher the particle size is, the higher the dissolution rates that have been mentioned. Furthermore, PPAd nanoparticles that had the highest particle mean diameter showed the highest raloxifene HCl release rates, while PESu that had the lowest particle mean diameter showed the lowest drug release rates as well. These conclusions are at variance with earlier studies for drug loaded nanoparticles, which showed that the low paricle size of the nanoparticles enhances the dissolution properties of poorly soluble drugs [48,49,50,51,52,53,54,55,56,57,58]. However we believe that bigger nanoparticles absorb higher amounts of drug in their surface and so the burst effect is higher for the bigger-sized nanoparticles. Except for the initial burst effect we found that the release rate is slightly higher for the bigger nanoparticles. This can be explained by the melting points of the polymers. The polymers that shape bigger nanoparticles are the copolymers with higher content in PAd which have lower melting points. The macromolecular chains of polymers with lower melting points have higher mobility in the dissolution temperature conditions and therefore higher amount of drug can be released.

## 3. Experimental

### 3.1. Materials

Succinic acid (SA) (99 %), adipic acid (99 %) and ethylene glycol (EG) (purum 99 percent) were purchased from Aldrich Chemical Co. 1,3-Propanediol (PD, Purity: > 99.7 %) was kindly supplied by Du Pont de Nemours Co. Tetrabutyl titanate (TBT) used as catalyst, was of analytical grade and it was purchased from Aldrich Chemical Co. Polyphosphoric acid (PPA) used as heat stabilizer was supplied by Fluka. *Rhizopus delemar* lipase and *Pseudomonas Cepacia* lipase were purchased from BioChemika. Raloxifene HCl drug was purchased from SOLMAG S.p.A (Milano Italy) as a yellowish crystalline powder with molecular mass 510.05, assay 99.4%, melting point 260 ^o^C and water content 0.602%. All the other materials and solvents used for the analytical methods were of analytical grade.

### 3.2. Synthesis of P(ESu-co-PAd) copolyesters

**Figure 12:**
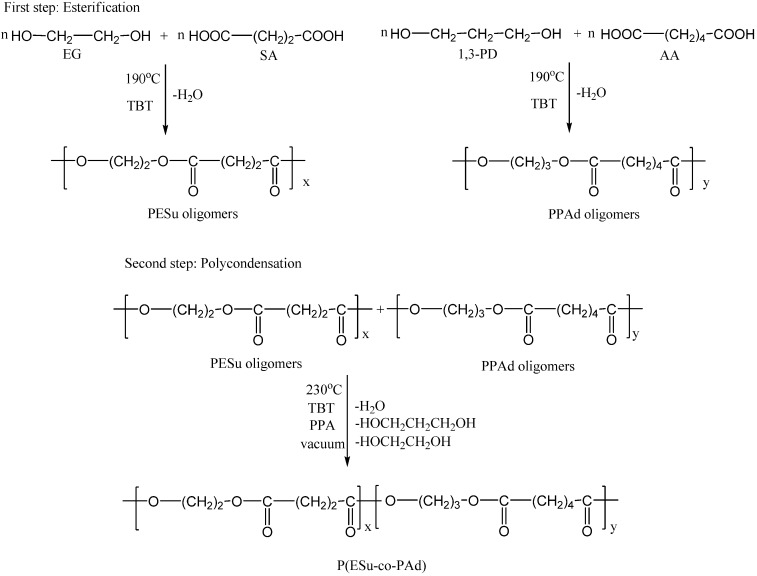
Synthetic route to P(ESu-co-PAd) copolymers preparation, via the two step polycondensation.

Synthesis of aliphatic copolyesters was performed following the two-stage melt polycondensation method (esterification and polycondensation) in a glass batch reactor [14]. At the first stage the oligomers were prepared (Figure 12). In brief, the proper amount of succinic acid or adipic acid and appropriate glycols in a molar ratio 1/1.2 and the catalyst TBT (3x10^-4^ mol TBT/mol SA) were charged into the reaction tube of the polycondensation apparatus. The apparatus with the reagents was evacuated several times and filled with argon in order to remove all the oxygen. The reaction mixture was heated at 190^o^C under an argon atmosphere and stirred at a constant speed (500 rpm). This first step (esterification) is considered to complete after the collection of theoretical amount of H_2_O, which was removed from the reaction mixture by distillation and collected in a graduated cylinder.

In the second polycondensation step the appropriate amounts of oligo(ethylene succinate) and oligo(propylene adipate) to synthesize the P(ESu-co-PAd) copolymers with the desired molar ratio were weighed. In this stage PPA, which is believed to prevent side reactions such as etherification and thermal decomposition, was added (5 x 10^-4^ mol PPA/mol SA). A vacuum (5.0 Pa) was applied slowly over a period of about 30 min, to avoid excessive foaming and to minimise oligomer sublimation, which is a potential problem during the melt polycondensation. The temperature was slowly increased to 230^o^C, while stirring speed was also increased to 720 rpm. The polycondensation continued for about 60 min for all prepared polyesters. After the end of the polycondensation reaction, the polyesters that had been crystallized were easily removed, milled and washed with methanol. 

### 3.3. Polymer characterization

#### 3.3.1. Intrinsic viscosity

Intrinsic viscosity [η] measurements were performed using an Ubbelohde viscometer at 25 ^o^C in chloroform. All polyesters were dissolved at room temperature in order to prepare solutions of 1 percent by weight and filtered through a disposable membrane filter 0.2 μm (Teflon). 

#### 3.3.2. End group analysis

Carboxyl end-group content (C.C.) of the resins about 0.1 g of polyesters was dissolved in chloroform at room temperature and the solution was titrated using a standard NaOH solution in methanol (N/10) and phenol red as indicator.

#### 3.3.3. Enzymatic hydrolysis

Polyesters in the form of films 2 x 3 cm in size and approximately 0.4 mm thickness, prepared by melt-pressing using a hydraulic press, were placed in Petri dishes containing phosphate buffer solution (pH 7.2) with 0.09 mg/mL *Rhizopus delemar* lipase and 0.01 mg/mL *Pseudomonas cepacia* lipase. The Petri dishes were then incubated at 37±1^o^C in an oven for several days while the media were replaced every 3 days. After a specific period of incubation (24 h), the films were removed from the Petri, washed with distilled water and weighted until constant weight. The degree of biodegradation was estimated from the mass loss.

#### 3.3.4. Polarizing Light Microscopy (PLM)

A polarizing light microscope (Nikon, Optiphot-2) equipped with a Linkam THMS 600 heating stage, a Linkam TP 91 control unit, and also a Jenoptic ProgRes C10Plus camera with the Capture Pro 2.1 software was used for PLM observations.

#### 3.3.5. Nuclear Magnetic Resonance (NMR)

^1^H-NMR spectra of polyesters were obtained with a Bruker spectrometer operating at a frequency of 400 MHz for protons. Deuterated chloroform was used as solvent in order to prepare solutions of 5 % w/v. The number of scans was 10 and the sweep width was 6 kHz.

### 3.4. Biocompatibility study of the prepared polyesters

#### 3.4.1. Cell culture

Human umbilical vein endothelial cells (HUVEC) were grown routinely in RPMI-1640 medium supplemented with 15 % fetal bovine serum (FBS), 15 mg ECGS, 100 U/mL penicillin, 100 μg/mL streptomycin, 50 μg/mL gentamycin and 2.5 μg/ml amphotericin B. Cultures were maintained at 37^o^C, 5 % CO_2_ and 100 % humidity.

#### 3.4.2. *In vitro* biocompatibility study

The biocompatibility of aliphatic polyesters, in comparison to biocompatible PLA, was evaluated by measuring the viability of HUVEC cells in the presence of different concentrations of the polymers. Cell viability was determined by the MTT assay. HUVEC cells were seeded in 24-well plates at a density of 30.000 cells per well in 500 μL cell culture medium. Twenty-four hours after plating, different amounts of aliphatic polyesters in the form of nanoparticles (suspended in water) were added in the wells. After 24 hours of incubation at 37^o^C, 50 μL of MTT solution (5 mg/mL in PBS pH 7.4) were added into each well and plates were incubated at 37^o^C for 2 hours. The medium was withdrawn and 200 μL acidified isopropanol (0.33 ml HCl in 100 mL isopropanol) were added in each well and agitated thoroughly to dissolve the formazan crystals. The solution was transferred to 96-well plates and immediately read on a microplate reader (Biorad, Hercules, CA, USA), at a wavelength of 490 nm. The experiments were performed in triplicate. Biocompatibility of polymers was expressed as percentage of cell viability, which was calculated from the ratio between the number of cells treated with the nanoparticles and that of non-treated cells (control).

### 3.5. Solubility measurements of Raloxifene HCl

Raloxifene HCl solubility was studied using pharmaceutical accepted solvents, such as acetone, ethanol, chloroform and dichloromethane. All the efforts to dilute the drug were carried out using a sonicator system at the same time. Raloxifene HCl characterization included Wide Angle X-Ray Diffractometry (WAXD) and Fourier Transformed-Infrared Spectroscopy (FT-IR), as well.

### 3.6. Preparation of nanoparticles

The nanoparticles were prepared by a variation of coprecipitation method. There are a lot of nanoparticle preparation methods [25], but for poorly water soluble drugs coprecipitation is a very simple and effective method. The drug raloxifene HCl (5 mg) was dissolved in 2 mL of the solvent system acetone/water 15/1 v/v using a sonicator for 1 min. Polyester was dissolved (50 mg) in the same solvent by sonication. The prepared solution was then transferred in portions to water (10 mL) in 30 min. The system was gently stirred until the evaporation of the organic solvent was complete. The nanoparticles were collected after being filtered by a microfilter with pore size of 1.2 μm (Millex AP, Millipore) in order to remove polymer aggregates and finally were freeze dried.

### 3.7. Characterisation of nanoparticles

#### 3.7.1. Wide Angle X-Ray Diffractometry (WAXD)

WAXD was used for the identification of the crystal properties of the pure materials and dispersions. WAXD study was performed over the range 2θ of 5 to 50^ο^C, using a Philips PW 1710 diffractometer with Bragg-Brentano geometry (θ, 2θ) and Ni-filtered CuKa radiation.

#### 3.7.2. Fourier Transformed-Infrared Spectroscopy (FT-IR)

FTIR spectra were obtained using a Perkin-Elmer FTIR spectrometer, model Spectrum 1000. A small amount of each material was mixed with KBr (1 wt% content) and compressed to tablets. The IR spectra of these tablets were obtained in absorbance mode and in the spectral region of 450 to 4,000 cm^-1^ using a resolution of 4 cm^-1^ and 64 co-added scans.

#### 3.7.3. Scanning Electron Microscopy (SEM)

The morphology of the prepared solid dispersions was examined by a scanning electron microscopy system (SEM) Jeol (JMS-840). The samples were covered with carbon coating in order to increase conductivity of the electron beam. Operating conditions were: accelerating voltage 20 kV, probe current 45 nA and counting time 60 seconds.

#### 3.7.4. Size measurements of nanoparticles

The particle size distribution of raloxifene HCl nanoparticles was determined by dynamic light scattering (DLS) using a Zetasizer Nano Instrument (Malvern Instruments, Nano ZS, ZEN3600, UK) operating with a 532 nm laser. A suitable amount of nanoparticles was dispersed in distilled water creating a total concentration 1 ‰ and was kept at 37^o^C under agitation at 100 rpm.

#### 3.7.5. Nanoparticles yield, drug loading content and entrapment efficiency

The obtained micellar solutions were frozen and lyophilized with a freeze drier system to obtain the dried nanoparticle products. Quantitative analysis was carried out by concentration determination, which was performed using UV detection at a wavelength 287 nm; previously an appropriate calibration curve was created. For the quantitative analysis the nanoparticles were washed with methanol and were left over night, in order to extract the drug from the nanoparticles. The nanoparticle yield, drug loading and drug entrapment efficiency were determined from equations (1)-(3) respectively:

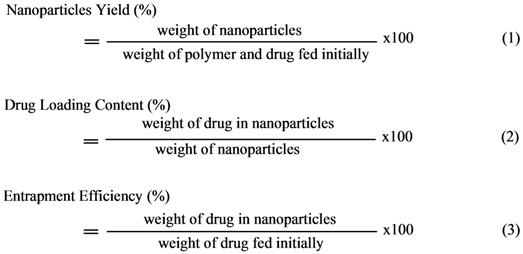


### 3.8. Dissolution testing

A Distek 2100C USP (baskets) type dissolution apparatus I was used. The dissolution testing was performed at 37±0.5 ºC and in one stage; the dissolution medium was 500 mL pH=7.4 and included Tween 20 (1.5 % vol.) in order to avoid sinking of the raloxifene HCl. The solubility of the drug in the dissolution medium is about 13 μg∙mL^−1^. All samples were collected using a Distek Evolution 4300 automatic sampler, filtered with nylon filters (Wattman 0.45 μm) and analyzed immediately after sampling, according to an appropriate HPLC method. Each test was performed in triplicate and the RSD was found to be less than 3 %. The analyses were performed using a Shimadzu Prominence HPLC system consisting of a DGU-20A5 degasser, LC-20 AD liquid chromatograph, SIL-20AC auto sampler, SPD-20A UV/Vis detector and CTO-20AC column oven. The column used for the separation was an NC-04 (250 x 4.0 mm), Nucleosil 100-5-CN 5.0 μm. The mobile phase was potassium dihydrogen phosphate (pH=3):acetonitrile (60:40 v/v), and the analytes were detected at 287 nm. The flow rate of the mobile phase was 1 mL/min and the column temperature was 40 °C.

## 4. Conclusions

Biodegradable random copolyesters P(ESu-co-PAd) were synthesized and used as drug carriers in nanoencapsulation treatment for the drug raloxifene HCl. The nanoparticles were prepared by a variation of the coprecipitation method. From the characterization of the copolyesters it was found that only P(ESu-co-PAd) 90/10, 80/20 and 70/30 were in crystalline form, while all the others remained amorphous. The melting point of the P(ESu-co-PAd) 70/30 is lower from the melting point of PPAd. These two polymers can be characterized as thermosensitive polymers and could be appropriate for targeting delivery systems. In terms of preparing raloxifene HCl nanoparticles it is the first time that such nanoparticles have been prepared. WAXD showed that raloxifene HCl is entrapped in crystalline form and possibly in nanocrystalline shapes within the nanoparticles. FTIR spectroscopy showed that there are no interactions between drug and the carriers used. The particle size distribution showed that the nanoparticles are in the range of 200-350 nm. It was found that the size of the nanoparticles is higher for the polymers with higher content in PAd. The drug release rates from the prepared polyesters are very low. It seems that initially the drug that was adsorbed in the surface of the nanoparticles was released, followed by very slow drug release rates for all the polyesters. It seems that these results depend on the drug’s crystallinity within the nanoparticles as well as on the melting point of used polyesters. Finally, it was found that nanoparticles with higher particle size mentioned higher release rates.
